# Comparison of DWI techniques in patients with epidermoid cyst: TGSE-BLADE DWI vs. SS-EPI DWI

**DOI:** 10.1007/s11604-024-01717-x

**Published:** 2024-12-28

**Authors:** Sayo Otani, Yasutaka Fushimi, Sachi Okuchi, Akihiko Sakata, Takayuki Yamamoto, Satoshi Nakajima, Yang Wang, Satoshi Ikeda, Shuichi Ito, Sumika Yasumura, Shigeki Takada, Noritaka Sano, Kentaro Ueno, Yuta Urushibata, Kun Zhou, Yoshiki Arakawa, Yuji Nakamoto

**Affiliations:** 1 Department of Diagnostic Imaging and Nuclear Medicine, Graduate School of Medicine, Kyoto University, 54 Shogoin Kawaharacho, Kyoto, 6068507 Japan; 2 Department of Neurosurgery, Graduate School of Medicine, Kyoto University, Kyoto, Japan; 3 Department of Biomedical Statistics and Bioinformatics, Graduate School of Medicine, Kyoto University, Kyoto, Japan; 4 Siemens Healthcare K. K., Tokyo, Japan; 5 Siemens Shenzhen Magnetic Resonance Ltd., Shenzhen, China

**Keywords:** Epidermoid cyst, Diffusion-weighted imaging, Turbo gradient- and spin-echo DWI with non-Cartesian BLADE trajectory, Single-shot echo-planar imaging, TGSE

## Abstract

**Purpose:**

To compare quantitative values and image quality between single-shot echo-planar imaging (SS-EPI) diffusion-weighted imaging (DWI) and two-dimensional turbo gradient- and spin-echo DWI with non-Cartesian BLADE trajectory (TGSE-BLADE DWI) in patients with epidermoid cyst.

**Methods:**

Patients with epidermoid cyst who underwent both SS-EPI DWI and TGSE-BLADE DWI were included in this study. Two raters placed ROIs encircling the entire epidermoid cyst on SS-EPI DWI, and then on TGSE-BLADE DWI. Apparent diffusion coefficient (ADC) of the epidermoid cyst was measured within each ROI, then the intraclass correlation coefficient (ICC) between raters was obtained for each DWI. The areas of ROIs placed by the two raters were measured and compared using the Dice coefficient. In the selected slice analysis, one rater selected the most appropriate slice and carefully placed the ROIs slightly smaller than the epidermoid outline to avoid artifacts. Image quality analysis was assessed qualitatively for geometric distortion, susceptibility artifacts, lesion conspicuity, and diagnostic confidence. ADCs for both DWI techniques were compared with theoretical values derived from the diffusion phantom.

**Results:**

Twenty patients with epidermoid cyst were included in this study. The ICC of ADC measured by the two raters for TGSE-BLADE (0.80) was higher than that for SS-EPI (0.59). Dice coefficient of ROI areas was significantly higher with TGSE-BLADE (0.78) than with SS-EPI (0.71, *P* = 0.007). Selected slice analysis showed that the ADC of epidermoid cyst was significantly higher with TGSE-BLADE DWI than with SS-EPI DWI (*P* < 0.001). ADCs measured from carefully selected ROIs avoiding artifacts with the two techniques correlated positively (*r* = 0.87, *P* < 0.001; ICC 0.75). TGSE-BLADE DWI rated better for image quality than SS-EPI DWI according to all raters. ICCs of measured ADC and theoretical ADCs exceeded 0.99 for both techniques.

**Conclusions:**

TGSE-BLADE DWI appears more suitable than SS-EPI DWI for evaluating epidermoid cyst.

**Supplementary Information:**

The online version contains supplementary material available at 10.1007/s11604-024-01717-x.

## Introduction

Epidermoid cyst arises from ectodermal inclusion during neural tube closure in the 3rd–5th week of embryogenesis. Epithelial cell rests may be transplanted to regions such as the cerebellopontine angle by the laterally migrating otic capsule or developing neurovasculature [1]. Epidermoid cyst contains both cholesterol and keratin, showing prolonged T1 and T2 relaxation times on MRI. Diffusion-weighted imaging (DWI) shows hyperintensity, which can help distinguish epidermoid cyst from surrounding cerebrospinal fluid. However, this hyperintensity on DWI is thought to represent a T2 shine-through effect rather than diffusion restriction of the epidermoid cyst [2]. Apparent diffusion coefficient (ADC) of the epidermoid cyst measured with a shorter diffusion time is higher than that measured with a longer diffusion time, reflecting the spatial restriction of water diffusion in the multiple keratin layers within the cyst as demonstrated by histopathology [3]. Although DWI is an essential MRI technique for evaluating epidermoid cyst, single-shot echo-planar imaging (SS-EPI) DWI, as the most widely used EPI-based DWI technique, is prone to susceptibility artifacts in areas of B0 field inhomogeneity, such as near air-bone interfaces and metallic implants [4, 5].

Many DWI techniques have been developed to overcome the distortion caused by magnetic susceptibility. These include field mapping corrections [6, 7], multi-shot EPI [8, 9], readout-segmented EPI (RESOLVE DWI) [10–12], point-spread function (PSF) encoding [13, 14], reversed gradient EPI (RG-EPI) [15], reverse encoding distortion correction EPI [16, 17], and zoomed EPI [18]. Turbo spin echo (TSE) has also been introduced, such as periodically rotated overlapping parallel lines with enhanced reconstruction (PROPELLER) DWI [19] and SS-TSE [20].

Among the various techniques as mentioned above, PROPELLER DWI, which is a TSE sequence with a non-Cartesian BLADE trajectory [21], has the advantage of reduced sensitivity to B0 inhomogeneity and robustness to patient motion due to oversampling of the central k-space [21]. However, the PROPELLER sequence has seen relatively little clinical application because of the long imaging time and high specific absorption rate (SAR) [22, 23]. Turbo gradient- and spin-echo with non-Cartesian BLADE trajectory (TGSE-BLADE) DWI has been introduced recently, and TGSE-BLADE DWI can be performed with a more clinically feasible acquisition time than PROPELLER due to its multi-blade k-space filling strategy [23, 24]. The SAR is decreased in TGSE-BLADE DWI due to the use of gradient echoes and reduction in the number of refocusing RF pulses [22–25]. Decreased geometric distortion and susceptibility artifacts have been reported with TGSE-BLADE DWI in previous studies such as investigations of cerebellopontine angle tumors [23], sinonasal lesions [26], cholesteatoma [27], aneurysmal clip [28] and cerebral infarction [29]. However, whether TGSE-BLADE DWI can reduce artifacts and improve image quality in patients with epidermoid cyst remains unknown.

The purpose of this study was to compare quantitative values and image quality between SS-EPI DWI and TGSE-BLADE DWI in patients with epidermoid cyst.

## Materials and methods

### Patients

Twenty patients with epidermoid who had undergone MRI including SS-EPI DWI and a prototype TGSE-BLADE DWI between December 2019 and October 2021 were included in this study. Two patients were excluded because the lesions were too small to evaluate.

This retrospective study was performed in accordance with the Declaration of Helsinki and was approved by Kyoto University Graduate School and Faculty of Medicine, Ethics Committee and the need to obtain informed consent was waived.

### Image acquisition

All patients underwent brain MRI using a 3-T scanner (MAGNETOM Prisma or Skyra; Siemens Healthineers, Erlangen, Germany) with a 64-channel head/neck coil or a 32-channel head coil. Two DWI sequences (SS-EPI DWI and TGSE-BLADE DWI) were acquired along with T2-weighted imaging (T2WI). SS-EPI DWI used: repetition time (TR), 5000 (5500) ms; echo time (TE), 53 (77) ms; field of view (FOV), 220 × 220 mm; matrix, 320 × 320; 35 slices; parallel imaging factor, GeneRalized Autocalibrating Partial Parallel Acquisition (GRAPPA) 3 × ; voxel size, 0.7 × 0.7 × 3 mm; diffusion time, 21.2 (27.4) ms; and acquisition time, 1 min 47 s. As a prototype sequence, TGSE-BLADE DWI used: TR, 7000 (7700) ms; TE, 46 (61) ms; flip angle (FA), 120°; FOV, 220 × 220 mm; matrix, 320 × 320; 35 slices; parallel imaging factor, N/A; voxel size, 0.7 × 0.7 × 3 mm; diffusion time, 17.6 (24.9) ms; and acquisition time, 3 min 53 s (4 m 16 s). Note that the values in parentheses are those used with the MAGNETOM Skyra (Table 1).

**Table 1:** Detailed imaging parameters for SS-EPI DWI and TGSE-BLADE DWI

Imaging parameter	SS-EPI DWI	TGSE-BLADE DWI
b value (s/mm^2^)	0, 1000	0, 1000
TR (ms)	5000, 5500*	7000, 7700*
TE (ms)	53, 77*	46, 61*
FA (degrees)	NA	120
FOV (mm^2^)	220 × 220	220 × 220
Matrix	320 × 320	160 × 160
Slice thickness (mm)	3	3
Number of slices	35	35
Voxel size (mm^3^)	0.7 × 0.7 × 3.0	1.4 × 1.4 × 3.0
Bandwidth (Hz/pixel)	1202	520
NEX	4	1
Parallel imaging	GRAPPA 3 ×	NA
Turbo factor	NA	13
EPI factor	128	3
Readout segments	NA	NA
Acquisition time	1 min 47 s	3 min 53 s, 4 min 16 s*

*TR* repetition time, *TE* echo time, *FA* flip angle, *FOV* field of view, *NEX* number of excitations, *EPI* echo planar imaging

Note that * represents the parameters for Skyra

T2WI was acquired in the same slice position with 2 DWIs, and the details of T2WI were as follows: axial acquisition; TR, 3200 ms; TE, 79 ms; FA, 120°; FOV, 185–199 × 220 mm; matrix, 378–406 × 448; 35 slices; parallel imaging factor, 2; voxel size, 0.5 × 0.5 × 3 mm; and acquisition time, 1 min 44–50 s. T2WI was used as the reference in this study.

Other image sequences were also obtained for clinical scans but were not used for this study.

### Image analysis

The following image analyses were performed for SS-EPI DWI and TGSE-BLADE DWI.

#### (a) Epidermoid cyst

(1) Entire lesion analysis. Two board-certified radiologists (S.I. and Y.W., with 9 and 10 years of experience in neuroradiology, respectively) separately placed ROIs encircling the epidermoid using ImageJ version 1.53e (https://imagej.nih.gov/ij/). First, each radiologist placed ROIs over the entire epidermoid lesions in all slices on trace-weighted SS-EPI DWIs without referring to any other image sequences and saved the ROI files independently. ROIs on TGSE-BLADE DWI were placed in the same manner 3 weeks later to avoid any learning effect.

ADC of epidermoid cyst was measured with the ROIs of each rater on SS-EPI DWI and TGSE-BLADE DWI, then the intraclass correlation coefficient (ICC) between two raters was obtained for each DWI.

The areas of ROIs placed by the two raters were measured and compared using the Dice coefficient, measured using the following equation where |A| represents the area of the ROI from Rater 1, and |B| represents the area of the ROI from Rater 2 [30]. |A ∩ B| represents the overlapping area of the ROIs from Raters 1 and 2.

$${\text{Dice coefficient}}\, = \,\frac{{\left| {{\text{A}} \cap \left. {\text{B}} \right| \times 2} \right.}}{{|{\text{A}}| + |{\text{B}}|}}$$

As the equation indicates, a Dice coefficient of 1 indicates that the ROIs of the two raters are identical, and 0 indicates no overlap. Dice coefficient was obtained for SS-EPI DWI and TGSE-BLADE DWI, respectively.

(2) Selected slice analysis. To evaluate the difference in ADC between SS-EPI and TGSE-BLADE DWIs, one board-certified radiologist (S.O., with 13 years of experience in neuroradiology) selected the most appropriate slice and carefully placed the ROIs slightly smaller than the epidermoid outline to avoid artifacts in SS-EPI and TGSE-BLADE DWIs (juxta-marginal ROIs). ADC for the carefully selected slice was analyzed.

#### (b) Qualitative evaluation

Image quality of the trace-weighted DWI of each sequence type was qualitatively assessed for geometric distortion, susceptibility artifacts, lesion conspicuity, diagnostic confidence, and overall image quality using a 4-point Likert scale [23]. The criteria for image assessment are defined in Supplementary Table 1. The assessment was conducted by three board-certified radiologists (S.I., Y.W., and S.O. with 9, 10, and 13 years of experience in neuroradiology, respectively).

#### (c) Phantom study

The phantom study was performed on a Mini Diffusion phantom (CaliberMRI, Boulder, CO, USA) with a MAGNETOM Prisma or Skyra system using the corresponding receiver coil. The phantom contains three high-performance liquid chromatography water vials (reference), and 10 polyvinylpyrrolidone (PVP) vials (two each of 10%, 20%, 30%, 40%, and 50% PVP). SS-EPI DWI and TGSE-BLADE DWI were scanned 4 times, respectively. ROIs were placed in the trace DWI images and superposed onto ADC maps. The accuracy of SS-EPI DWI and TGSE-BLADE DWI was evaluated by comparison between measured and theoretical ADCs corresponding to the temperature of the phantom.

### Statistical analysis

The relationship between ADCs of SS-EPI and TGSE-BLADE was assessed using Pearson’s correlation coefficient (*r*) if both values were normally distributed and Spearman’s rank correlation coefficient (*ρ*) if one or both values was not normally distributed. Normal distribution was evaluated using the Shapiro–Wilk test. Agreement among ADCs in the selected slice analysis from SS-EPI and TGSE-BLADE was assessed by the ICC. Dice coefficient of the area of the ROI for SS-EPI DWI and TGSE-BLADE DWI was compared using the paired *t*-test. The McNemar test was used by three raters to analyze image quality. The accuracy of ADC in the phantom study was evaluated by ICC.

Values of *P* < 0.05 were considered significant. Statistical analysis was performed using JMP Pro version 16.0 software (SAS Institute Inc., Cary, NC, USA) and MedCalc version 16.2.0 (MedCalc Software, Ostend, Belgium). Continuous variables are presented as medians and interquartile ranges unless otherwise noted.

## Results

### Patients

**Fig. 1:**
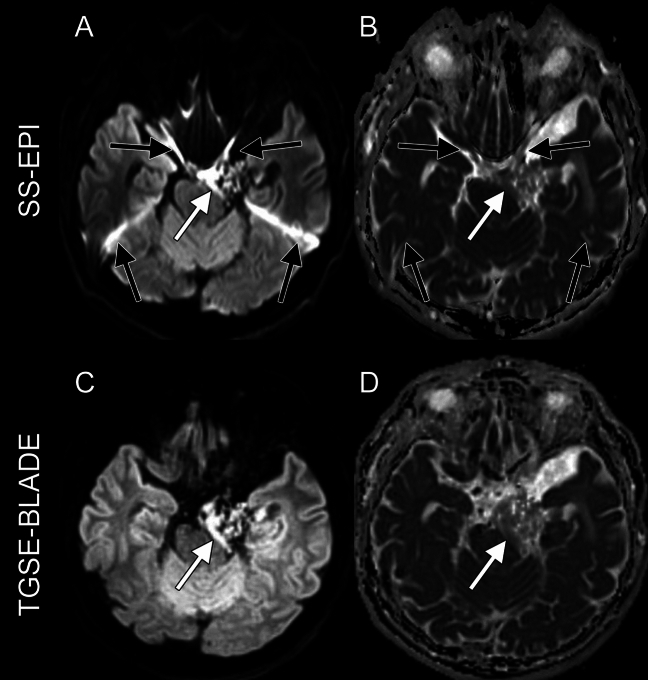
Representative images of a 62-year-old man with post-operative status of left cerebellopontine angle epidermoid cyst: b1000 and ADC from SS-EPI DWI (**A**, **B**) and from TGSE-BLADE DWI (**C**, **D**) are shown. Black arrows indicate susceptibility artifacts. Artifacts are seen near air-bone interfaces on SS-EPI DWI affecting the visualization of residual epidermoid cyst (white arrow), whereas few artifacts are seen on TGSE-BLADE DWI

Twenty patients (12 females, 8 males; median age, 57 years; range, 19–78 years) were included. The demographics of all participants are shown in Table 2. Figure 1 shows representative images of SS-EPI DWI and TGSE-BLADE DWI in patients with epidermoid cyst.

**Table 2:** Demographics of subjects

Age [years]	57 [19–78]
Sex	Female, 12
	Male, 8
Diagnosis	Pathological, 15
	Clinically suspected, 5
Region	Cerebellopontine angle, 14
	Fourth ventricle, 2
	Lateral ventricle, 1
	Interpeduncular cistern, 1
	Ambient cistern, 1
	Frontal bone, 1

Note that “Region” represents the main area occupied by the epidermoid cyst

#### (a) Epidermoid cyst

**Fig. 2:**
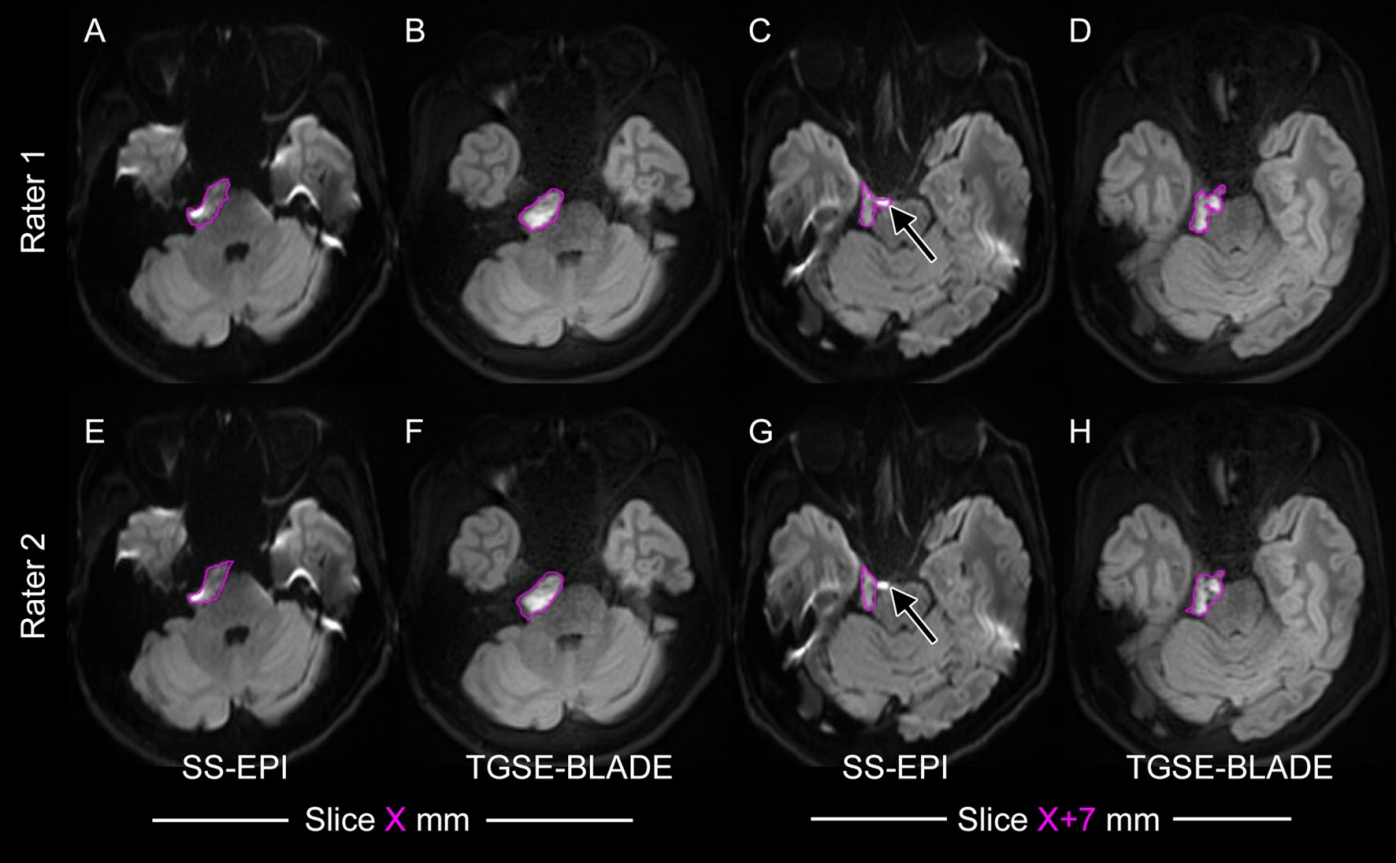
Representative images of the suspected epidermoid cyst at the right cerebellopontine angle. Rater 1 (**A**, **B**, **C**, **D**) and Rater 2 (**E**, **F**, **G**, **H**) independently placed ROIs encircling the entire epidermoid lesions on SS-EPI DWIs (**A**, **C**, **E**, **G**). Subsequently, Rater 1 and Rater 2 placed ROIs in the same manner on TGSE-BLADE DWI (**B**, **D**, **F**, **H**) 3 weeks later to avoid any learning effect. ROIs for the entire lesion analysis (Slice X mm and Slice X + 7 mm) are shown (magenta). Rater 1 included a hyperintense area in front of the pons on SS-EPI DWI (arrow, C) as the lesion, while Rater 2 did not (arrow, G). A major discrepancy in ROIs between raters was observed on SS-EPI DWI. These ROIs created by Rater 1 and Rater 2 were also used for Dice coefficient analysis

**Fig. 3:**
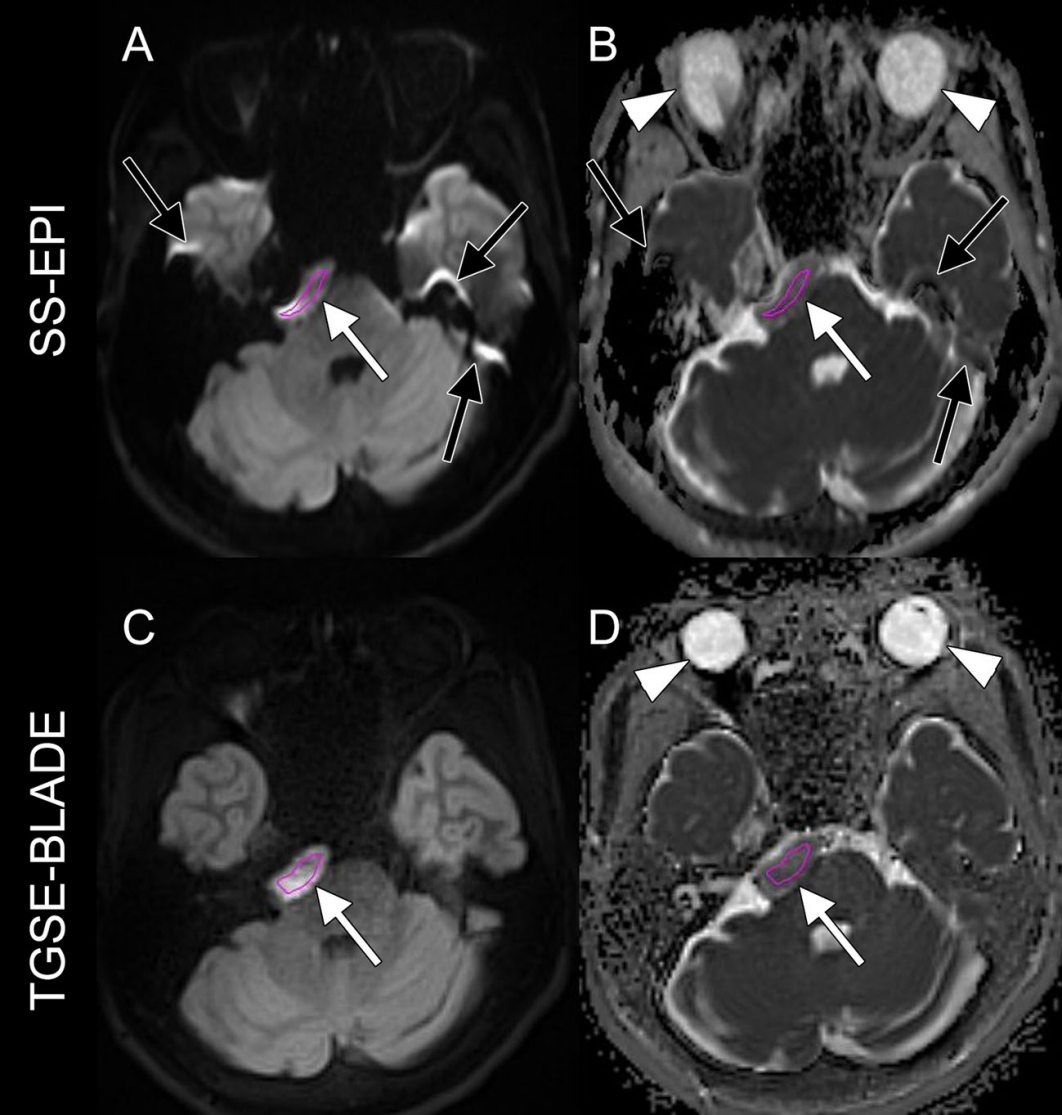
Another rater selected a representative slice in which the lesion was well recognized on both DWI techniques, and carefully placed medium-size ROIs for the epidermoid cyst while avoiding artifacts on b1000 images using SS-EPI and TGSE-BLADE DWI (white arrows) **A**, **C**) Juxta-marginal ROIs. These ROIs were used for ADC calculation (white arrows, **B**, **D**). Note that multiple susceptibility artifacts are shown on SS-EPI DWI (black arrows, **A**, **B**). The shape of the eyeballs is deformed on SS-EPI (arrowhead, B), but remains circular on TGSE-BLADE (arrowhead, **D**)

(1) Entire lesion analysis. Representative ROIs provided by two raters are shown in Fig. 2. The ICC of ADC measured by two raters was higher for TGSE-BLADE (0.80) than for SS-EPI (0.59). Dice coefficient of ROI areas was significantly higher for TGSE-BLADE (0.78) than for SS-EPI (0.71; *P* = 0.007).

(2) Selected slice analysis. Another rater selected the most appropriate slice and carefully placed medium-sized ROIs for the epidermoid cyst while avoiding artifacts on SS-EPI and TGSE-BLADE DWI (Fig. 3). ADC of epidermoid cyst was 1204.4 ± 204.6 × 10^–6^ mm^2^/s for SS-EPI DWI and 1323.8 ± 195.8 × 10^–6^ mm^2^/s for TGSE-BLADE DWI. Mean ADC was significantly higher for TGSE-BLADE DWI than for SS-EPI DWI (*P* < 0.001). ADCs measured from carefully selected ROIs avoiding artifacts with each DWI technique showed a positive correlation (*r* = 0.87, *P* < 0.001), and the ICC was 0.75 (95% confidence interval [CI] 0.05–0.92).

#### (b) Image quality study

Geometric distortion, susceptibility artifacts, lesion conspicuity, diagnostic confidence and overall imaging quality were better in TGSE-BLADE DWI compared with SS-EPI DWI in all raters (Table 3).

**Table 3:** Results of image quality for SS-EPI DWI and TGSE-BLADE DWI

Rater 1	SS-EPI DWI	TGSE-BLADE DWI	*P* value
Distortion	2 (2–2)	4 (4–4)	< 0.001
Artifact	2 (2–2)	4 (4–4)	< 0.001
Conspicuity	2 (2–2)	4 (3–4)	< 0.001
Diagnostic confidence	2 (2–2)	4 (3–4)	< 0.001
Overall image	2 (2–2)	4 (4–4)	< 0.001

Rater 2	SS-EPI DWI	TGSE-BLADE DWI	*P* value
Distortion	3 (2–3)	4 (4–4)	0.003
Artifact	3 (2–3)	4 (4–4)	0.005
Conspicuity	3 (2.25–3)	4 (4–4)	0.03
Diagnostic confidence	3 (2–3)	4 (4–4)	0.01
Overall image	3 (2–3)	4 (4–4)	0.01

Rater 3	SS-EPI DWI	TGSE-BLADE DWI	*P* value
Distortion	2 (2–3)	4 (4–4)	< 0.001
Artifact	2 (2–3)	4 (4–4)	< 0.001
Conspicuity	2 (2–3)	4 (4–4)	< 0.001
Diagnostic confidence	3 (3–3)	4 (4–4)	0.08
Overall image	3 (2–3)	4 (4–4)	0.005

Note that data are presented as the median (interquartile range). The McNemar test was used for statistical analysis

#### (c) Phantom study

**Fig. 4:**
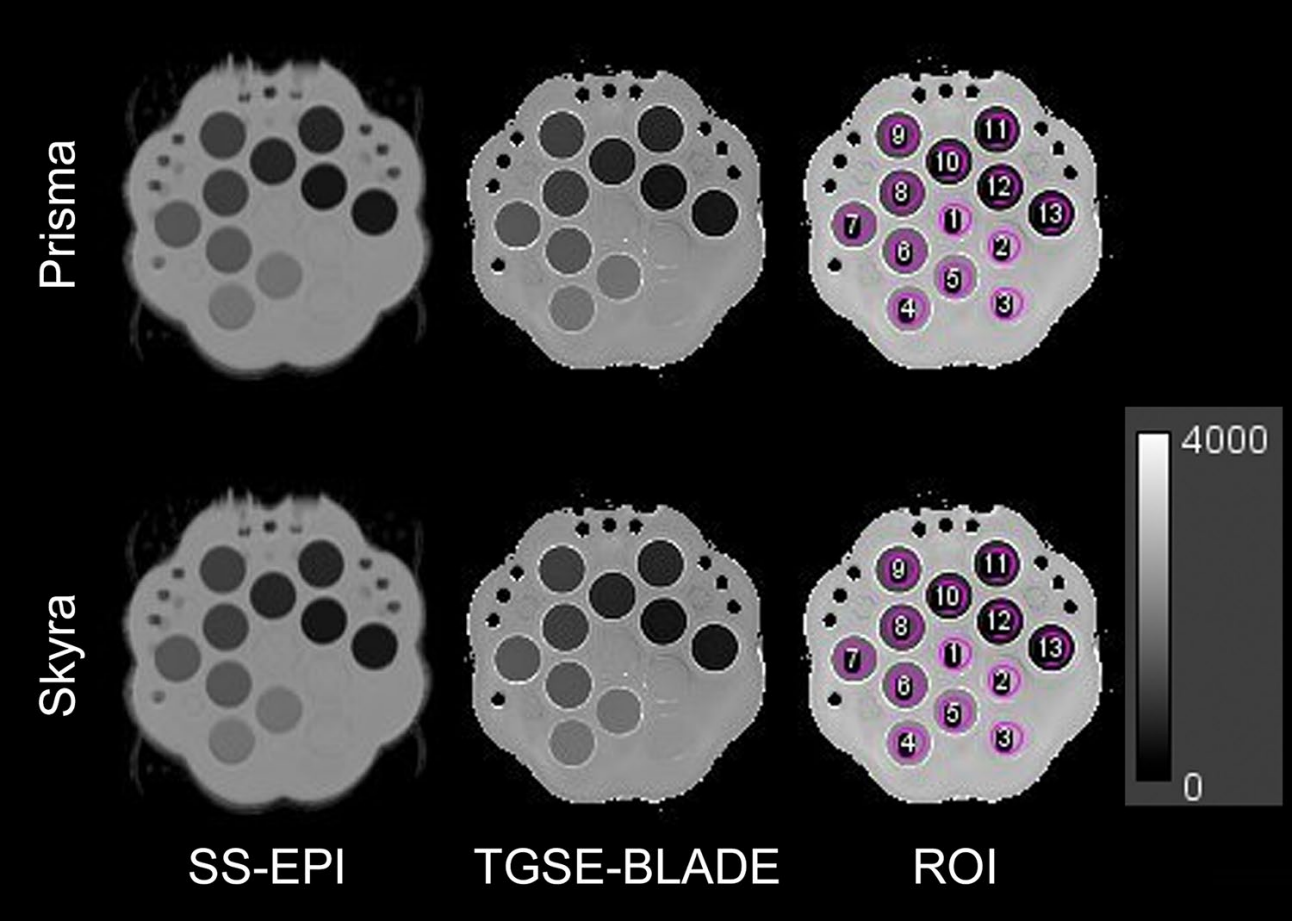
The diffusion phantom was scanned using the MAGNETOM Prisma and Skyra. SS-EPI and TGSE-BLADE DWI were performed 4 times each. ADCs and theoretical ADCs corresponding to the temperature of the phantom were compared. Note that the phantom contains three high-performance liquid chromatography water vials (ROI 1, 2, and 3), 10% PVP (ROI 4, 5), 20% PVP (ROI 6, 7), 30% PVP (ROI 8, 9), 40% PVP (ROI 10, 11), and 50% PVP (ROI 12, 13). The grayscale bar represents ADC [× 10^–6^ mm^2^/s]. PVP, polyvinylpyrrolidone

The scan was performed under a temperature of 24.0 ℃ (Fig. 4). Measured ADC and theoretical ADC were shown Table 4. ICCs of measured ADC were as follows: Prisma, SS-EPI, 0.9998 [0.9993, 1.0000], TGSE-BLADE, 0.9997 [0.9982, 0.9999]; Skyra, SS-EPI, 0.9980 [0.9760, 0.9995], TGSE-BLADE, 0.9924 [0.5870, 0.9986].

**Table 4:** Theoretical and measured ADCs [10^–6^ mm^2^/s]

		Theoretical Value	Measured ADCs			
			Prisma		Skyra	
			SS-EPI	TGSE-BLADE	SS-EPI	TGSE-BLADE
ROI 1	Water	2225	2249.0 ± 6.4	2231.0 ± 4.9	2252.0 ± 1.6	2225.4 ± 3.1
ROI 2	Water	2225	2268.0 ± 2.3	2215.7 ± 5.4	2234.7 ± 2.6	2255.8 ± 0.4
ROI 3	Water	2225	2252.0 ± 4.5	2251.8 ± 2.9	2346.3 ± 3.0	2363.9 ± 4.1
ROI 4	PVP10	1727	1783.0 ± 1.4	1753.9 ± 2.2	1855.2 ± 1.5	1858.6 ± 0.5
ROI 5	PVP10	1727	1760.0 ± 3.0	1745.3 ± 3.3	1821.0 ± 1.4	1812.8 ± 2.4
ROI 6	PVP20	1313	1339.0 ± 4.5	1329.7 ± 2.9	1397.2 ± 1.5	1383.0 ± 1.7
ROI 7	PVP20	1313	1349.0 ± 2.0	1338.6 ± 1.8	1430.8 ± 0.9	1427.3 ± 0.7
ROI 8	PVP30	960	980.0 ± 1.9	965.1 ± 1.1	1037.9 ± 2.6	1028.9 ± 0.7
ROI 9	PVP30	960	979.0 ± 2.2	976.1 ± 1.6	1078.3 ± 1.5	1059.5 ± 1.6
ROI 10	PVP40	585	608.0 ± 3.3	590.5 ± 1.7	661.9 ± 3.8	653.9 ± 2.9
ROI 11	PVP40	585	606.0 ± 6.6	601.3 ± 1.6	696.7 ± 3.4	689.5 ± 3.2
ROI 12	PVP50	356	366.0 ± 0.7	342.0 ± 2.5	395.9 ± 4.2	374.7 ± 4.0
ROI 13	PVP50	356	375.0 ± 1.4	354.6 ± 1.7	421.6 ± 3.5	432.1 ± 1.8

Theoretical ADCs of vials at a temperature of 24.0 °C are shown

## Discussion

SS-EPI DWI and TGSE-BLADE DWI were compared for both the entire lesion and selected slices of epidermoid cyst, and image quality of both DWI techniques was also evaluated in this study. ADCs for both techniques were compared with theoretical values derived from the diffusion phantom.

The concordance of ADCs obtained from ROIs encompassing the epidermoid cyst placed by the two raters was higher for TGSE-BLADE than for SS-EPI in the entire-lesion analysis. Dice coefficient was also higher for TGSE-BLADE than for SS-EPI. A possible reason for this is that epidermoid cysts tend to occur at the skull base where susceptibility artifacts are commonly seen due to the presence of skull bone and of air in the paranasal sinus and mastoid air cells [31], meaning that the entire tumor is often difficult to delineate with SS-EPI DWI. TGSE-BLADE DWI suffers from fewer distortion artifacts, which contributed to the higher inter-rater ADC concordance. Further, in this study, Dice coefficient was used as a score indicating the concordance of regions designated as ROIs by the raters. The significantly higher concordance of the ROI-designated regions in TGSE-BLADE DWI supports higher ADC concordance with TGSE-BLADE.

Selected slice analysis was performed to avoid artifacts and achieve as much commonality as possible. ADC of epidermoid cyst between SS-EPI and TGSE-BLADE was found to correlate very well, and in terms of less artifacts on TGSE-BLADE, ADC of TGSE-BLADE can be used clinically. However, the ADC of the epidermoid cyst was higher for TGSE-BLADE than for SS-EPI. A possible reason for this is that the diffusion time of TGSE-BLADE was slightly shorter than that of SS-EPI, leading to slightly different ADCs derived from these two DWIs. ADC may become slightly higher with shorter diffusion time probably due to spatial restriction of water diffusion in the multiple keratin layers within the epidermoid cyst [3]. The diffusion phantom study demonstrated no major differences in ADCs between SS-EPI and TGSE-BLADE, probably because the vials in the diffusion phantom comprised PVP and water solutions, and lacked the complex layer structures of epidermoid cyst in addition to less susceptibility artifact.

Image quality analysis demonstrated that geometric distortion, susceptibility artifacts, lesion conspicuity, diagnostic confidence, and overall image quality were better in TGSE-BLADE DWI compared with SS-EPI DWI. These results were consistent with the findings from previous reports [23, 26–28]. We therefore believe that TGSE-BLADE DWI is useful for diagnosing epidermoid cyst due to the reduced image distortion and fewer susceptibility artifacts.

Several limitations to this study need to be kept in mind. First, this investigation included only a small number of patients. Second, we evaluated image quality and distortion only using 3-T MRI. Because the severity of the image artifact shows a non-linear relationship with field strength, image artifacts should be evaluated at each field strength [32]. Finally, the acquisition time in TGSE-BLADE DWI is clinically acceptable, but still much longer than that in SS-EPI DWI. To avoid signal-to-noise ratio (SNR) loss, we did not use any acceleration techniques (such as parallel imaging) in TGSE-BLADE DWI. In clinical practice, utilizing an appropriate acceleration factor that can balance acquisition time and SNR might be worthwhile.

In conclusion, the concordance of ADCs for epidermoid cyst obtained by raters was higher for TGSE-BLADE than for SS-EPI, and the concordance of ROIs obtained for TGSE-BLADE DWI was also higher than that for SS-EPI. Although ADC of epidermoid cyst was slightly higher for TGSE-BLADE than for SS-EPI, both ADCs correlated very well. Moreover, SS-EPI and TGSE-BLADE showed good concordance with theoretical ADCs provided by the diffusion phantom. TGSE-BLADE DWI appears more suitable than SS-EPI DWI as a tool for evaluating epidermoid cyst.

## Supplementary Information

Below is the link to the electronic supplementary material.

Supplementary file1 (DOCX 17 KB)
