# Supplementary material for: Comparison of DWI techniques in patients with epidermoid cyst: TGSE-BLADE DWI vs. SS-EPI DWI
Source: Jpn J Radiol. 2024 Dec 28;43(5):752–60. doi: 10.1007/s11604-024-01717-x (PMC12053340; doi:10.1007/s11604-024-01717-x)
Supplement: Supplementary file 1 — Supplementary file1 (DOCX 17 KB) [file 11604_2024_1717_MOESM1_ESM.docx]

**Supplemental Table 1**

| Grade | Geometric  distortion | Susceptibility artifacts | Lesion  conspicuity | Diagnostic confidence | Overall image  quality |
| --- | --- | --- | --- | --- | --- |
| 1 | Severe distortion | Severe artifacts | Unable to evaluate | Vague (10–39%) | Poor, insufficient for diagnosis |
| 2 | Moderate distortion | Major artifacts | Acceptable for visualization | Likely (40–69%) | Fair, adequate for diagnosis |
| 3 | Mild distortion | Only minor artifacts | Obvious visibility | High (70–89%) | Good for diagnosis |
| 4 | No distortion | No artifacts | Excellent for visualization | Definite (90–100%) | Excellent for diagnosis |
